# Supplementary material for: Hazard-based distributional regression via ordinary differential equations
Source: Stat Methods Med Res. 2026 Jan 19;35(3):571–87. doi: 10.1177/09622802251412840 (PMC13047225; doi:10.1177/09622802251412840)
Supplement: sj-pdf-1-smm-10.1177_09622802251412840 - Supplemental material for Hazard-based distributional regression via ordinary differential equations [file sj-pdf-1-smm-10.1177_09622802251412840.pdf]

Journal Title
XX(X):1–18
©The Author(s) 2016
Reprints and permission:
sagepub.co.uk/journalsPermissions.nav
DOI: 10.1177/ToBeAssigned
www.sagepub.com/
SAGE

Appendix: "Hazard-based distributional regression via ordinary differential equations"

J. Andres Christen[1] and F. Javier Rubio[2]

Keywords
Distributional regression; Hazard function; ODE solver; Ordinary differential equations; Survival analysis.

Supplementary Material

1 Technical conditions

Recall that the hazard function $h(t \mid \boldsymbol{\eta}, \mathbf{x}_i)$ and the cumulative hazard function $H(t \mid \boldsymbol{\eta}, \mathbf{x}_i)$ are retrieved as solutions to the system of ODEs (2). Note that the density function $f(t \mid \boldsymbol{\eta}, \mathbf{x}_i)$ can be obtained as $f(t \mid \boldsymbol{\eta}, \mathbf{x}_i) = h(t \mid \boldsymbol{\eta}, \mathbf{x}_i) \exp\{-H(t \mid \boldsymbol{\eta}, \mathbf{x}_i)\}$, the cumulative distribution function as $F(t \mid \boldsymbol{\eta}, \mathbf{x}_i) = 1 - \exp\{-H(t \mid \boldsymbol{\eta}, \mathbf{x}_i)\}$, and the survival function $S(t \mid \boldsymbol{\eta}, \mathbf{x}_i) = \exp\{-H(t \mid \boldsymbol{\eta}, \mathbf{x}_i)\}$.

For the case where the initial conditions $\mathbf{Y}_0$ are fixed, the log-likelihood function is:

$$\begin{aligned}\ell_n(\boldsymbol{\eta}) = \sum_{i=1}^n \ell_i(\boldsymbol{\eta}) &= \sum_{i=1}^n \delta_i \log h(t_i \mid \boldsymbol{\eta}, \mathbf{x}_i) - \sum_{i=1}^n H(t_i \mid \boldsymbol{\eta}, \mathbf{x}_i) \\ &= \sum_{i=1}^n \delta_i \log f(t_i \mid \boldsymbol{\eta}, \mathbf{x}_i) + \sum_{i=1}^n (1-\delta_i) \log S(t_i \mid \boldsymbol{\eta}, \mathbf{x}_i),\end{aligned}$$

From the below assumptions on the initial value problem (IVP) (2), the gradient of the log-likelihood $\nabla_{\boldsymbol{\eta}} \ell_n(\boldsymbol{\eta})$ exist and is continuous. Define $Q_n(\boldsymbol{\eta}) = [\nabla_{\boldsymbol{\eta}} \ell_n(\boldsymbol{\eta})] [\nabla_{\boldsymbol{\eta}} \ell_n(\boldsymbol{\eta})]^\top$, then by the weak law of large numbers $\frac{1}{n} Q_n(\boldsymbol{\eta}) \stackrel{\Pr}{\to} Q(\boldsymbol{\eta}) = \mathbb{E}\left\{ [\nabla_{\boldsymbol{\eta}} \ell_i(\boldsymbol{\eta})] [\nabla_{\boldsymbol{\eta}} \ell_i(\boldsymbol{\eta})]^\top \right\}$, as $n \to \infty$, for each value of $\boldsymbol{\eta}$, since

[1]Department of Statistics, Centre for Research in Mathematics (CIMAT). Guanajuato, Mexico
[2]Department of Statistical Science, University College London. London, UK

Corresponding author:
F. Javier Rubio Department of Statistical Science, University College London. London, UK
Email: f.j.rubio@ucl.ac.uk

each partial derivative of $\ell_n(\boldsymbol{\eta})$ is a summation of n terms. The function $Q(\boldsymbol{\eta})$, which consists of the cross-products of all partial derivatives of the log-likelihood, can be seen as a generalised version of the standard Fisher information matrix (Hjort 1992), incorporating the censoring process.

Consider the following regularity conditions.

C1. The parameter space $\tilde{\Theta}$ is a compact subset of $\mathbb{R}^{\tilde{d}}$, and $\boldsymbol{\eta}^* \in \tilde{\Theta}$ is the true value of the parameter. Additionally, we assume that $t \in [0, \tau]$, for $\tau > 0$.

C2. The vector field $\psi_{\boldsymbol{\eta}} : D \to \mathbb{R}^{m+1}$, where $D \subseteq \mathbb{R}^{m+1}$ is a closed rectangle, is three-times continuously differentiable in $\mathbf{Y}$, three times continuously differentiable in $\boldsymbol{\eta}$, and $\mathbf{Y}_0$ is in the interior of D.

C3. The solution $h(t \mid \boldsymbol{\eta}, \mathbf{x})$ is identifiable. That is, if $h(t \mid \boldsymbol{\eta}_1, \mathbf{x}) = h(t \mid \boldsymbol{\eta}_2, \mathbf{x})$, for $t \in [0, \tau]$ and all $\mathbf{x}$, implies that $\boldsymbol{\eta}_1 = \boldsymbol{\eta}_2$.

C4. The matrix $Q(\boldsymbol{\eta}^*)$ is non-singular.

C5. The covariates $\mathbf{x}_i$ are independent and identically distributed. Let $\mathbf{X}_k$, $k = 1, \dots, d$, be the design matrix corresponding to the covariates included in the linear predictor for the kth parameter, θ_k, in the ODE system. Assume that there exists n_0 such that, for $n > n_0$, the matrices $\mathbf{X}_k^\top \mathbf{X}_k$ are positive definite almost surely, and $\frac{1}{n}\mathbf{X}_k^\top \mathbf{X}_k \overset{\Pr}{\to} \Sigma_k$, for some $k \times k$ positive definite matrix Σ_k, as $n \to \infty$.

C6. $0 < \mathbb{P}(\delta_i = 1) \leq 1$, and $O_i \perp C_i \mid \mathbf{x}_i$ (non-informative censoring conditional on covariates).

2 Theoretical results

Before presenting the proof of Propositon 1, we present two preliminary results that guarantee the standard regularity conditions under conditions C1-C3.

Lemma 1. *Suppose that conditions C1-C3 are satisfied. Then, for each value of $\mathbf{x}$, there exist functions $M_1(t, \mathbf{x})$ and $M_2(t, \mathbf{x})$ such that, for $i = 1, \dots, \tilde{d}$ and $j = 1, \dots, \tilde{d}$,*

$$\left| \frac{\partial \log f(t \mid \boldsymbol{\eta}, \mathbf{x})}{\partial \boldsymbol{\eta}_i} \cdot \frac{\partial \log f(t \mid \boldsymbol{\eta}, \mathbf{x})}{\partial \boldsymbol{\eta}_j} \right| \leq M_1(t, \mathbf{x}),$$
$$\left| \frac{\partial^2 \log f(t \mid \boldsymbol{\eta}, \mathbf{x})}{\partial \boldsymbol{\eta}_i \partial \boldsymbol{\eta}_j} \right| \leq M_2(t, \mathbf{x}),$$

where

$$\int M_1(t, \mathbf{x}) dF(t \mid \boldsymbol{\eta}, \mathbf{x}) < \infty,$$
$$\int M_2(t, \mathbf{x}) dF(t \mid \boldsymbol{\eta}, \mathbf{x}) < \infty.$$

Proof. Conditions C1-C3 $\psi_{\boldsymbol{\eta}}(\mathbf{Y}, \mathbf{x}_i)$ in (2) has continuous third partial derivatives with respect to $\mathbf{y}$ and the parameters $\boldsymbol{\eta}$ (C3), this guarantees existence and uniqueness of a solution of the initial value problem (IVP) (2). Moreover, the second partial derivatives of $\mathbf{Y}(t \mid \boldsymbol{\eta}, \mathbf{x}_i)$ and $H(t \mid \boldsymbol{\eta}, \mathbf{x}_i)$ with respect to the parameters $\boldsymbol{\eta}$ exist, and therefore the first derivatives are continuous. These results can be found, for example, in Jackiewicz (2009), Theorem 1.5.1, and can be extended by reapplying the theorem to the derivatives themselves. Thus, conditions C1-C3 guarantee that the solution to the IVP are twice continuously differentiable with respect to $\mathbf{Y}$ and the parameters $\boldsymbol{\eta}$. Note also that

$$\begin{aligned}\frac{\partial \log f(t \mid \boldsymbol{\eta}, \mathbf{x})}{\partial \boldsymbol{\eta}_i} &= \frac{\partial \log h(t \mid \boldsymbol{\eta}, \mathbf{x})}{\partial \boldsymbol{\eta}_i} - \frac{\partial H(t \mid \boldsymbol{\eta}, \mathbf{x})}{\partial \boldsymbol{\eta}_i} \\ &= \frac{\dfrac{\partial h(t \mid \boldsymbol{\eta}, \mathbf{x})}{\partial \boldsymbol{\eta}_i}}{h(t \mid \boldsymbol{\eta}, \mathbf{x})} - \frac{\partial H(t \mid \boldsymbol{\eta}, \mathbf{x})}{\partial \boldsymbol{\eta}_i},\end{aligned}$$

and

$$\frac{\partial^2 \log f(t \mid \boldsymbol{\eta}, \mathbf{x})}{\partial \boldsymbol{\eta}_i \boldsymbol{\eta}_j} = \frac{\dfrac{\partial^2 h(t \mid \boldsymbol{\eta}, \mathbf{x})}{\partial \boldsymbol{\eta}_i \partial \boldsymbol{\eta}_j} - \dfrac{\partial h(t \mid \boldsymbol{\eta}, \mathbf{x})}{\partial \boldsymbol{\eta}_i} \dfrac{\partial h(t \mid \boldsymbol{\eta}, \mathbf{x})}{\partial \boldsymbol{\eta}_j}}{h(t \mid \boldsymbol{\eta}, \mathbf{x})^2} - \frac{\partial H(t \mid \boldsymbol{\eta}, \mathbf{x})}{\partial \boldsymbol{\eta}_i}.$$

By condition C3, and since we are focusing on autonomous systems (2), it follows that the terms in the previous equations are continuous in t. Recalling that $t \in [0, \tau]$, it follows that these functions are Lebesgue integrable for each value of $\mathbf{x}$. Equivalently, there exist functions $M_1(t, \mathbf{x})$ and $M_2(t, \mathbf{x})$ such that

$$\left| \frac{\dfrac{\partial h(t \mid \boldsymbol{\eta}, \mathbf{x})}{\partial \boldsymbol{\eta}_i}}{h(t \mid \boldsymbol{\eta}, \mathbf{x})} - \frac{\partial H(t \mid \boldsymbol{\eta}, \mathbf{x})}{\partial \boldsymbol{\eta}_i} \right| \le M_1(t, \mathbf{x}),$$

$$\left| \frac{\dfrac{\partial^2 h(t \mid \boldsymbol{\eta}, \mathbf{x})}{\partial \boldsymbol{\eta}_i \partial \boldsymbol{\eta}_j} - \dfrac{\partial h(t \mid \boldsymbol{\eta}, \mathbf{x})}{\partial \boldsymbol{\eta}_i} \dfrac{\partial h(t \mid \boldsymbol{\eta}, \mathbf{x})}{\partial \boldsymbol{\eta}_j}}{h(t \mid \boldsymbol{\eta}, \mathbf{x})^2} - \frac{\partial H(t \mid \boldsymbol{\eta}, \mathbf{x})}{\partial \boldsymbol{\eta}_i} \right| \le M_2(t, \mathbf{x}),$$

where

$$\begin{aligned}\int M_1(t, \mathbf{x}) dF(t \mid \boldsymbol{\eta}, \mathbf{x}) &< \infty, \\ \int M_2(t, \mathbf{x}) dF(t \mid \boldsymbol{\eta}, \mathbf{x}) &< \infty.\end{aligned}$$

Using the equivalence of the derivatives of $\log f(t \mid \boldsymbol{\eta}, \mathbf{x})$ and those of $h(t \mid \boldsymbol{\eta}, \mathbf{x})$ and $H(t \mid \boldsymbol{\eta}, \mathbf{x})$, the result follows.

Now, we prove a lemma that shows that under some regularity conditions on the right-hand side, the survival regression models defined by the system of ODEs (2) is differentiable in quadratic mean.

Lemma 2. *Let $h(t \mid \boldsymbol{\eta}, \mathbf{x})$ be the hazard function obtained as the solution to the system of ODEs (2) for a given covariate $\mathbf{x}$. Suppose that conditions C1–C3 hold. Then, the model $f(t \mid \boldsymbol{\eta}, \mathbf{x})$ is differentiable in quadratic mean.*

Proof. First, note that condition C2 implies that the right-hand side $\Psi_{\boldsymbol{\eta}}(\mathbf{Y}(t \mid \boldsymbol{\eta}, \mathbf{x}), \mathbf{x})$ is Lipschitz continuous in $\mathbf{Y}$, and consequently there exists a unique solution to the autonomous system of ODEs (2) (see Chapter 4 from Hirsch et al. (2013)). Such solution is identifiable by assumption C3. The differentiability condition C2 guarantees that $h(t \mid \boldsymbol{\eta}, \mathbf{x})$ is twice continuously differentiable with respect to $\boldsymbol{\eta}$ (see Chapter 1 from Jackiewicz (2009)). These points, together with the above discussion of the differentiability of the log-likelihood, imply that the map $\boldsymbol{\eta} \mapsto \sqrt{f(t \mid \boldsymbol{\eta}, \mathbf{x})}$ is continuously differentiable for each $t > 0$.

Lemma 1 implies that the entries of the matrix $Q(\boldsymbol{\eta})$ are continuous in $\boldsymbol{\eta}$ (see Chapter 5 from Lehmann and Casella (2006)). These results together with Lemma 7.6 from Van der Vaart (2000) imply differentiability of the root density $\boldsymbol{\eta} \mapsto \sqrt{f(t \mid \boldsymbol{\eta}, \mathbf{x})}$ in quadratic mean. That is, for each $\mathbf{x}$

$$\int_0^\infty \left[\sqrt{f(t \mid \boldsymbol{\eta} + \mathbf{h}, \mathbf{x})} - \sqrt{f(t \mid \boldsymbol{\eta}, \mathbf{x})} - \frac{1}{2}\left(\mathbf{h}^\top \nabla_{\boldsymbol{\eta}} \log f(t \mid \boldsymbol{\eta}, \mathbf{x})\right) \sqrt{f(t \mid \boldsymbol{\eta}, \mathbf{x})}\right] dt = o\left(\|\mathbf{h}\|^2\right),$$

as $\|\mathbf{h}\| \to 0$.

Proof of Proposition 1. First, note that, by Theorem 7.2 in Van der Vaart (2000), we have that differentiability in square mean implies local asymptotic normality (LAN) when the sample is uncensored. Using the results (Example 7) in Le Cam and Yang (1988) and condition C6, it follows that LAN is preserved under non-informative right censoring.

Now, the restriction of the parameter space and t to compact sets in C1 implies the existence of uniformly consistent hypothesis tests (Van der Vaart 2000; Nickl 2013). That is, there exists a sequence of tests W_n for testing

$$H_0 : \boldsymbol{\eta} = \boldsymbol{\eta}^* \quad \text{vs.} \quad H_1 : \|\boldsymbol{\eta} - \boldsymbol{\eta}^*\| > \epsilon,$$

for every $\epsilon > 0$, which satisfy

$$\begin{aligned} \mathbb{E}_{\boldsymbol{\eta}^*}(W_n) &\to 0, \\ \sup_{\|\boldsymbol{\eta} - \boldsymbol{\eta}^*\| \geq \epsilon} \mathbb{E}_{\boldsymbol{\eta}}(1 - W_n) &\to 0, \quad \text{as} \quad n \to \infty. \end{aligned} \tag{1}$$

The proof of Proposition 1 follows from Lemma 2, which establishes differentiability in quadratic mean for the survival regression models; the argument above, which proves local asymptotic normality under censoring; the testing condition (1); and Theorem 10.3 from Van der Vaart (2000).■

3 MCMC sampler for Bayesian variable selection

In this section, we present the Gibbs sampler algorithm proposed for selecting the variables that enter each predictor of the distributional regression model. More advanced methods exist and could be seamlessly integrated with our methodology (Liang et al. 2023).

Algorithm 1 Gibbs sampler for Bayesian variable selection

1: Initialise.

- $\boldsymbol{\gamma}^{(0)} = \left(\gamma_{k,j}^{(0)}\right), j = 2, \dots, p_k, k = 1, \dots, d.$
- Compute $\widehat{p}\left(\mathbf{t}, \boldsymbol{\delta}, \mathbf{X} \mid \boldsymbol{\gamma}^{(0)}\right)$.
- Set iter $= 0$.

2: Iterate for iter $= 1, \dots, M$.
For each predictor k and each covariate j, update the inclusion matrix $\boldsymbol{\gamma}$ updating each $\gamma_{k,j}$, using its full conditional:

- Let $\gamma'_{k,j} = 1 - \gamma_{k,j}^{(t-1)}$, and define the new model matrix $\boldsymbol{\gamma}'$, where only the (k, j)-th entry has changed.
- Compute the probability

$$P = \frac{\widehat{p}\left(\mathbf{t}, \boldsymbol{\delta}, \mathbf{X} \mid \boldsymbol{\gamma}'\right) \pi(\boldsymbol{\gamma}')}{\widehat{p}\left(\mathbf{t}, \boldsymbol{\delta}, \mathbf{X} \mid \boldsymbol{\gamma}'\right) \pi(\boldsymbol{\gamma}') + \widehat{p}\left(\mathbf{t}, \boldsymbol{\delta}, \mathbf{X} \mid \boldsymbol{\gamma}^{(t-1)}\right) \pi(\boldsymbol{\gamma}^{(t-1)})}.$$

- Simulate from the full conditional, that is change to $\gamma'_{k,j}$ with probability P or remain with $\gamma_{k,j}^{(t-1)}$ with probability $1 - P$:
  - Draw $U \sim \text{Unif}(0, 1)$,
  - If $U < P$, set $\gamma_{k,j}^{(t)} = \gamma'_{k,j}$,
  - otherwise, keep $\gamma_{k,j}^{(t)} = \gamma_{k,j}^{(t-1)}$.

4 Simulation from the hazard-response regression model

The following algorithm presents a method to produce approximate simulations from the hazard-response regression model (5) – (8). This algorithm represents an extension to that proposed in (Christen and Rubio 2024) in the context without covariates.

In step 1, the solution to the system of ODEs can be obtained using the Julia library `DifferentialEquations.jl` and the `solve()` function. Similarly, step 2 can be implemented

Algorithm 2 Approximate Simulation from the Hazard-Response regression model (5) – (8)

For a grid of M time points $\tilde{\mathbf{t}}_i = \{t_{1i}, \dots, t_{Mi}\}$ in the interval $[0, t_{Mi}]$, and for each $i = 1, \dots, n$:

1. Obtain a numerical solution of the ODE system (5), with parameters $\boldsymbol{\eta}$ and covariate value $\mathbf{x}_i$ at $\tilde{\mathbf{t}}_i$, using an ODE Solver.
2. Using the grid $\tilde{\mathbf{t}}_i$ and the corresponding values of solution for the cumulative hazard function H evaluated at $\tilde{\mathbf{t}}_i$, construct an approximation $\tilde{H}^{-1}$, of $H^{-1}(\cdot \mid \boldsymbol{\eta}, \mathbf{x}_i)$.
3. Generate $u_i \sim U(0, 1)$.
4. Calculate the simulated value $t_i^* = \tilde{H}^{-1}\left(-\log(u_i)\right)$.

automatically using the output from `solve()`. When simulating data with right-censoring, the upper bound t_{Mi} can be set to the maximum follow-up time.

5 Simulation results

Parameter	mean (MAP)	median (MAP)	se (MAP)	RMSE (MAP)	width	coverage
$\beta_{1,0}$ (1.5)	1.528	1.526	0.042	0.051	0.240	0.960
$\beta_{1,1}$ (0.5)	0.499	0.499	0.054	0.054	0.215	0.922
$\beta_{2,0}$ (0.5)	0.453	0.451	0.055	0.073	0.301	0.972
$\beta_{2,1}$ (-0.5)	-0.497	-0.490	0.073	0.073	0.278	0.910
$\beta_{3,0}$ (1.0)	1.079	1.081	0.053	0.095	0.374	0.992
$\beta_{3,1}$ (0.5)	0.500	0.497	0.056	0.056	0.219	0.930
$\beta_{4,0}$ (3.0)	3.001	2.994	0.076	0.076	0.291	0.956
$\beta_{4,1}$ (-0.5)	-0.487	-0.478	0.070	0.072	0.271	0.908

Table 1. Simulation results: censoring rate 20%, $n = 500$.

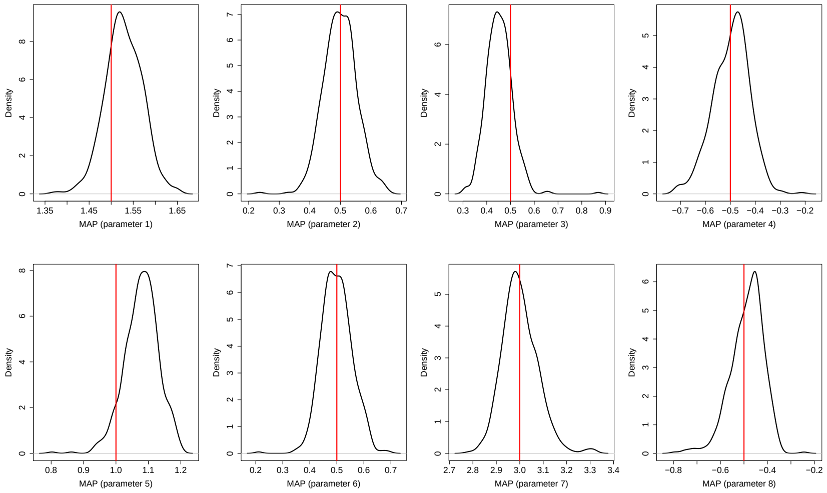

Figure 1. Simulation results: censoring rate 20%, $n = 500$.

Parameter	mean (MAP)	median (MAP)	se (MAP)	RMSE (MAP)	width	coverage
$\beta_{1,0}$ (1.5)	1.527	1.526	0.021	0.034	0.116	0.924
$\beta_{1,1}$ (0.5)	0.496	0.495	0.025	0.026	0.103	0.950
$\beta_{2,0}$ (0.5)	0.522	0.522	0.025	0.033	0.147	0.984
$\beta_{2,1}$ (-0.5)	-0.497	-0.496	0.033	0.033	0.133	0.950
$\beta_{3,0}$ (1)	1.005	1.005	0.026	0.027	0.185	0.998
$\beta_{3,1}$ (0.5)	0.498	0.495	0.026	0.026	0.104	0.950
$\beta_{4,0}$ (3)	2.999	2.996	0.037	0.037	0.139	0.940
$\beta_{4,1}$ (-0.5)	-0.485	-0.484	0.029	0.033	0.125	0.934

Table 2. Simulation results: censoring rate 20%, $n = 2000$.

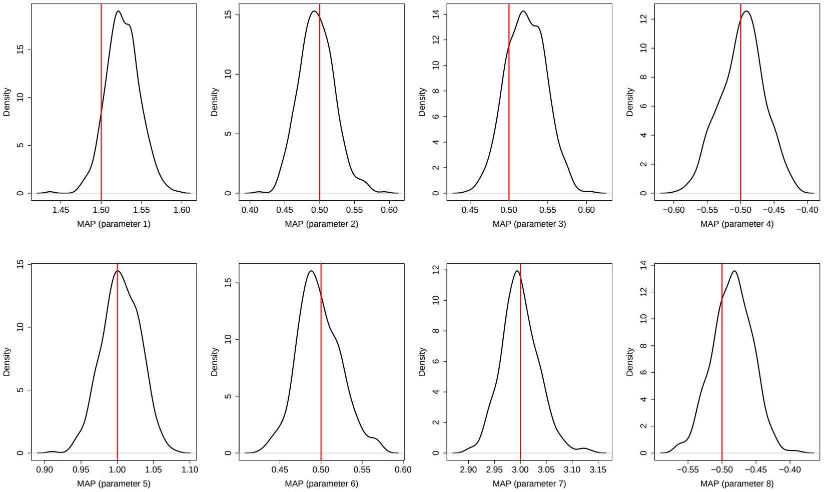

Figure 2. Simulation results: censoring rate 20%, $n = 2000$.

Parameter	mean (MAP)	median (MAP)	se (MAP)	RMSE (MAP)	width	coverage
$\beta_{1,0}$ (1.5)	1.529	1.528	0.046	0.054	0.249	0.972
$\beta_{1,1}$ (0.5)	0.499	0.499	0.065	0.065	0.252	0.934
$\beta_{2,0}$ (0.5)	0.458	0.457	0.094	0.103	0.423	0.956
$\beta_{2,1}$ (-0.5)	-0.498	-0.490	0.128	0.128	0.460	0.918
$\beta_{3,0}$ (1)	1.081	1.083	0.077	0.112	0.453	0.972
$\beta_{3,1}$ (0.5)	0.501	0.492	0.081	0.081	0.290	0.912
$\beta_{4,0}$ (3)	3.001	2.990	0.090	0.090	0.326	0.940
$\beta_{4,1}$ (-0.5)	-0.483	-0.469	0.092	0.094	0.331	0.886

Table 3. Simulation results: censoring rate 40%, $n = 500$.

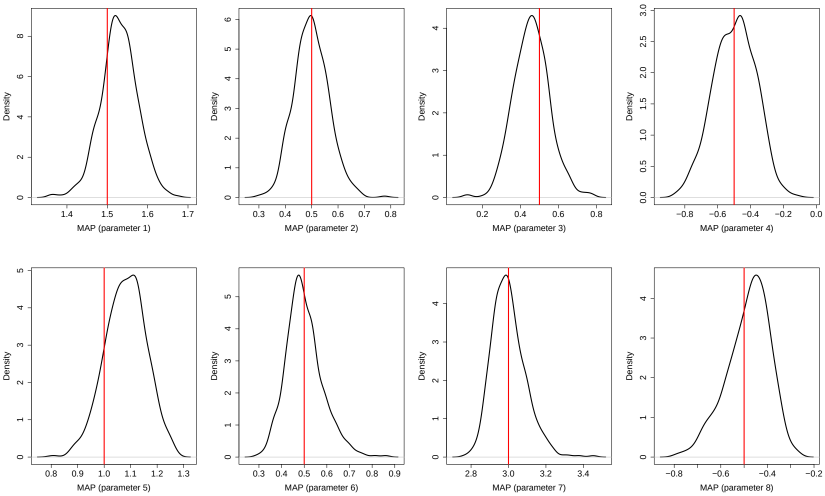

Figure 3. Simulation results: censoring rate 40%, $n = 500$.

Parameter	mean (MAP)	median (MAP)	se (MAP)	RMSE (MAP)	width	coverage
$\beta_{1,0}$ (1.5)	1.538	1.539	0.031	0.050	0.177	0.938
$\beta_{1,1}$ (0.5)	0.495	0.493	0.045	0.045	0.175	0.938
$\beta_{2,0}$ (0.5)	0.476	0.474	0.065	0.069	0.296	0.970
$\beta_{2,1}$ (-0.5)	-0.494	-0.488	0.088	0.088	0.320	0.908
$\beta_{3,0}$ (1)	1.069	1.073	0.053	0.087	0.321	0.968
$\beta_{3,1}$ (0.5)	0.492	0.489	0.049	0.049	0.197	0.952
$\beta_{4,0}$ (3)	3.006	3.002	0.056	0.057	0.222	0.960
$\beta_{4,1}$ (-0.5)	-0.479	-0.473	0.061	0.064	0.223	0.882

Table 4. Simulation results: censoring rate 40%, $n = 1000$.

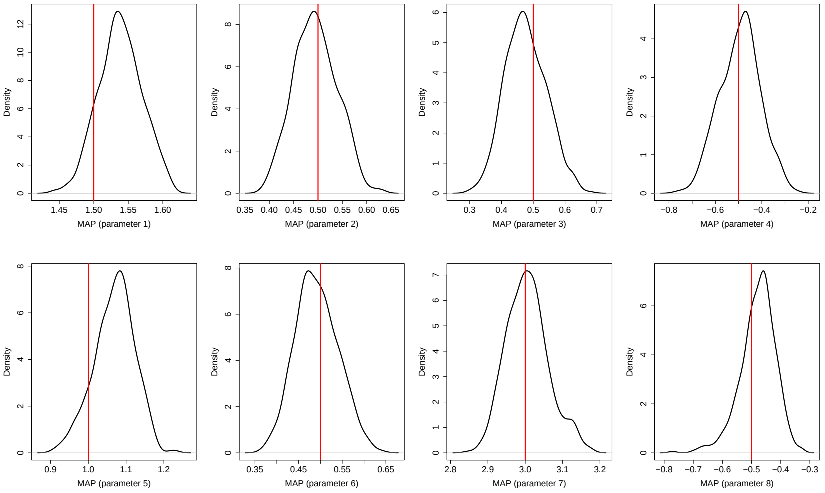

Figure 4. Simulation results: censoring rate 40%, $n = 1000$.

Parameter	mean (MAP)	median (MAP)	se (MAP)	RMSE (MAP)	coverage
$\beta_{1,0}$ (1.5)	1.525	1.524	0.022	0.033	0.934
$\beta_{1,1}$ (0.5)	0.498	0.497	0.031	0.031	0.940
$\beta_{2,0}$ (0.5)	0.530	0.532	0.043	0.053	0.934
$\beta_{2,1}$ (-0.5)	-0.497	-0.495	0.055	0.055	0.948
$\beta_{3,0}$ (1)	0.997	0.996	0.039	0.039	0.996
$\beta_{3,1}$ (0.5)	0.498	0.495	0.036	0.036	0.954
$\beta_{4,0}$ (3)	3.001	2.998	0.040	0.040	0.946
$\beta_{4,1}$ (-0.5)	-0.487	-0.484	0.039	0.041	0.916

Table 5. Simulation results: censoring rate 40%, $n = 2000$.

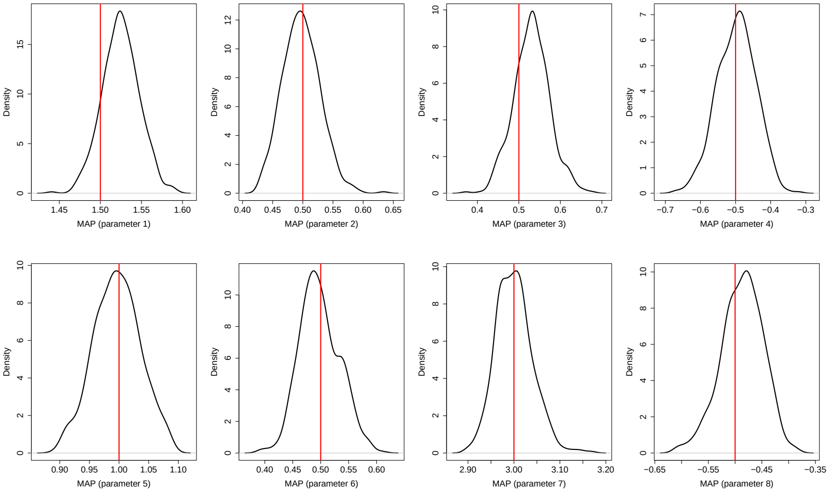

Figure 5. Simulation results: censoring rate 40%, $n = 2000$.

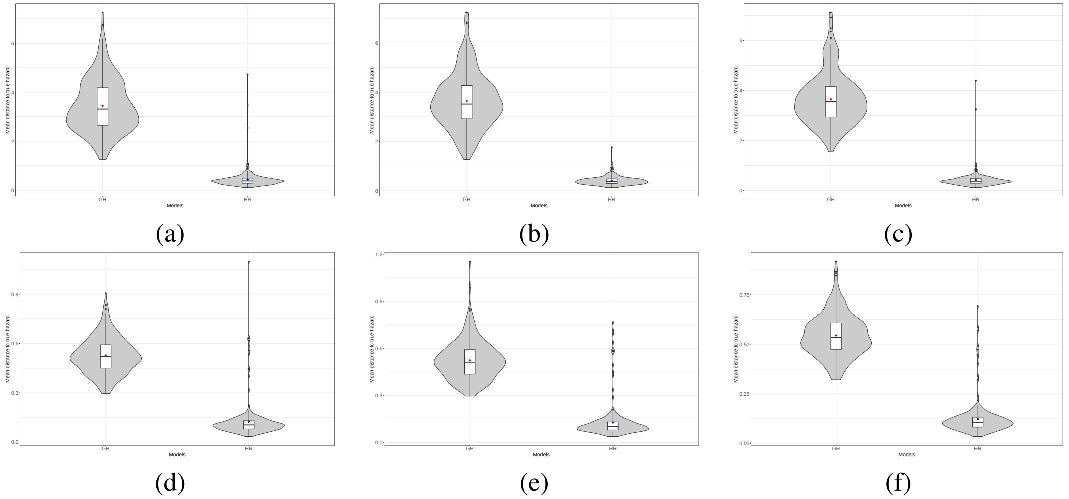

Figure 6. Mean hazard distance to the true hazard function: (a) $n = 500$, 20% censoring, (b) $n = 1000$, 20% censoring, (c) $n = 2000$, 20% censoring, (d) $n = 500$, 40% censoring, (e) $n = 1000$, 40% censoring, (f) $n = 2000$, 40% censoring.

6 Posterior summaries: Ipilimumab immunotherapy trial

This section presents posterior summaries for the Ipilimumab immunotherapy trial example presented in the main paper.

	α_0	α_1	β_0	β_1	h_0
Min.	-1.20	0.18	-2.68	-0.70	-7.22
1st Qu.	-0.04	0.93	-2.48	-0.41	-5.38
Median	0.11	1.17	-2.43	-0.35	-5.08
Mean	0.13	1.26	-2.43	-0.35	-5.13
3rd Qu.	0.29	1.45	-2.38	-0.28	-4.81
Max.	1.20	5.60	-2.13	-0.06	-3.36

Table 6. Ipilimumab immunotherapy trial data: Posterior summaries for the logistic growth model.

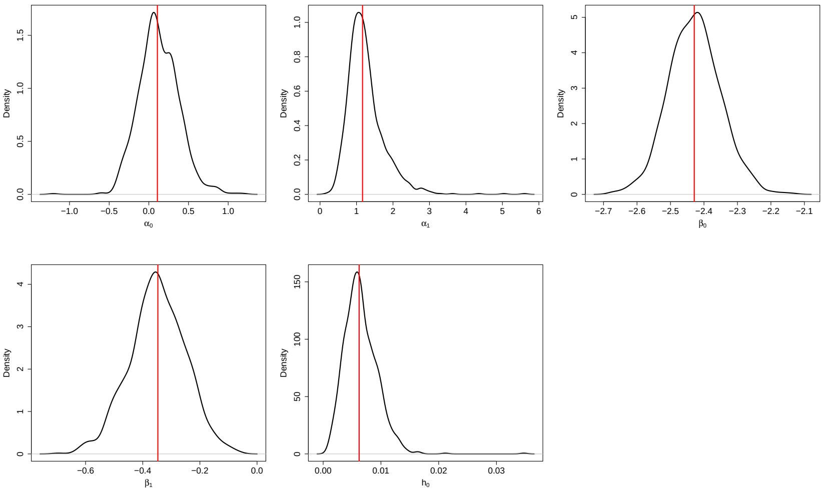

Figure 7. Ipilimumab immunotherapy trial data: Posterior samples. The posterior median in shown in the vertical red line.

7 Posterior summaries: Breast cancer recurrence

This section presents additional results for the Breast cancer recurrence application presented in the main paper.

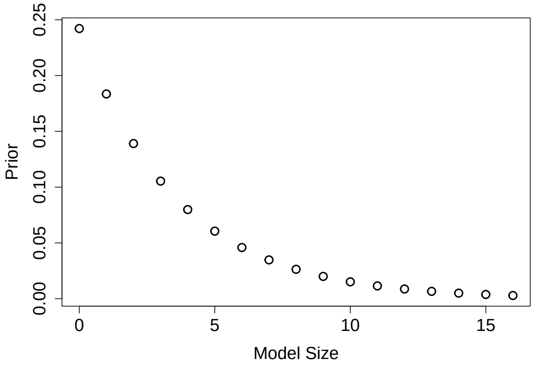

Figure 8. Complexity prior (normalised) $\pi(\gamma) \propto \tilde{d}^{-0.1|\gamma|}$.

	$\beta_{1,0}$	$\beta_{1,1}$	$\beta_{1,2}$	$\beta_{2,0}$	$\beta_{2,1}$	$\beta_{3,0}$	$\beta_{3,1}$	$\beta_{3,2}$	$\beta_{4,0}$	$\beta_{4,1}$	$\beta_{4,2}$	$\beta_{4,3}$
Min.	1.34	0.40	-1.11	-0.85	-0.59	0.53	0.48	-1.01	2.58	0.04	-0.43	-0.94
1st Qu.	1.49	0.50	-0.75	-0.01	-0.39	1.10	0.71	-0.69	2.98	0.10	-0.27	-0.57
Median	1.53	0.53	-0.66	0.19	-0.34	1.29	0.77	-0.62	3.05	0.12	-0.23	-0.48
Mean	1.53	0.53	-0.66	0.18	-0.34	1.29	0.77	-0.62	3.04	0.12	-0.23	-0.48
3rd Qu.	1.58	0.56	-0.58	0.38	-0.28	1.48	0.84	-0.54	3.12	0.13	-0.19	-0.39
Max.	1.81	0.77	-0.22	0.99	-0.07	2.11	1.05	-0.21	3.37	0.20	-0.05	-0.08

Table 7. Breast cancer recurrence data: Posterior summaries from the hazard-response model fitted via MCMC.

	$\beta_{1,0}$	$\beta_{1,1}$	$\beta_{1,2}$	$\beta_{2,0}$	$\beta_{2,1}$	$\beta_{3,0}$	$\beta_{3,1}$	$\beta_{3,2}$	$\beta_{4,0}$	$\beta_{4,1}$	$\beta_{4,2}$	$\beta_{4,3}$
Min.	1.23	0.42	-1.05	-0.95	-0.62	0.41	0.45	-1.01	2.71	0.02	-0.45	-0.87
1st Qu.	1.49	0.50	-0.72	0.03	-0.39	1.12	0.71	-0.68	3.00	0.10	-0.27	-0.54
Median	1.54	0.52	-0.64	0.20	-0.34	1.28	0.77	-0.61	3.06	0.11	-0.24	-0.46
Mean	1.54	0.52	-0.64	0.20	-0.34	1.28	0.77	-0.60	3.06	0.11	-0.24	-0.46
3rd Qu.	1.59	0.54	-0.57	0.37	-0.29	1.44	0.83	-0.53	3.11	0.13	-0.20	-0.38
Max.	1.80	0.64	-0.19	1.10	-0.05	2.34	1.12	-0.19	3.37	0.19	-0.04	-0.05
MAP	1.54	0.52	-0.64	0.20	-0.34	1.28	0.77	-0.61	3.06	0.11	-0.24	-0.46

Table 8. Breast cancer recurrence data: Posterior summaries from the hazard-response model using the normal approximation.

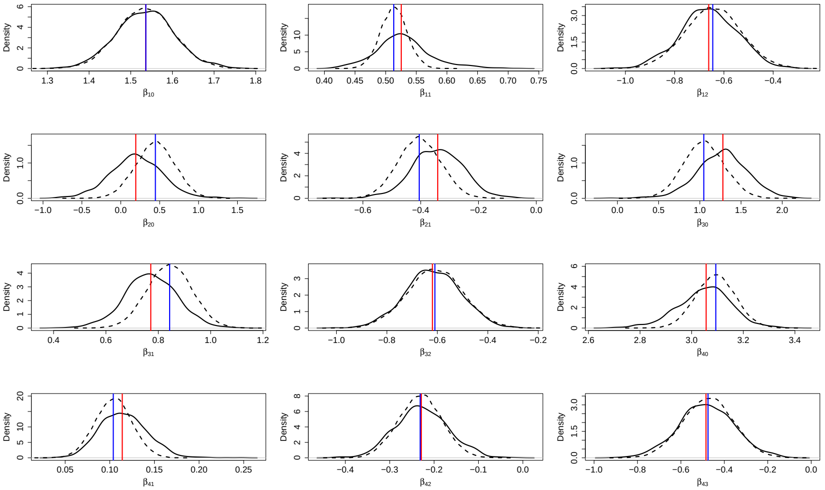

Figure 9. Breast cancer recurrence data: marginal kernel density estimators of the posterior samples using adaptive MCMC (solid line) and the normal approximation (dashed line). The posterior median from the MCMC sample is shown in the vertical red line and the MAP in blue line.

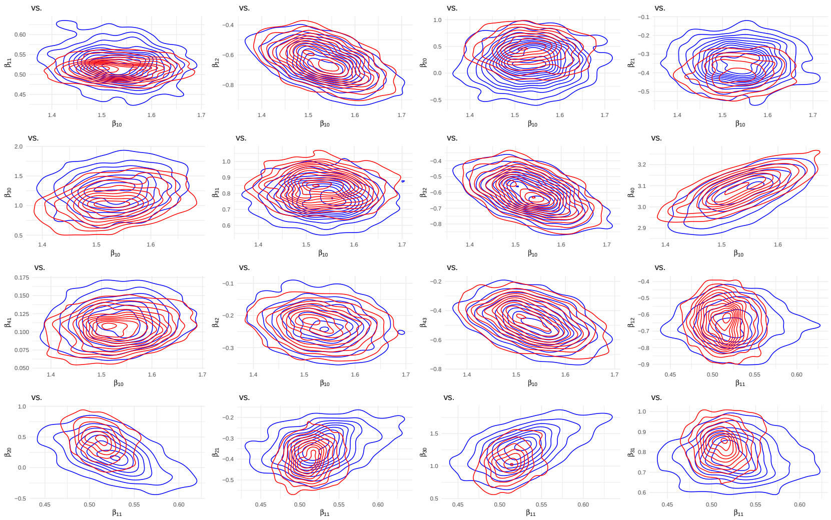

Figure 10. Breast cancer recurrence data: Contour plots of the 2D kernel density estimates for the MCMC samples (blue) and the normal approximation (red).

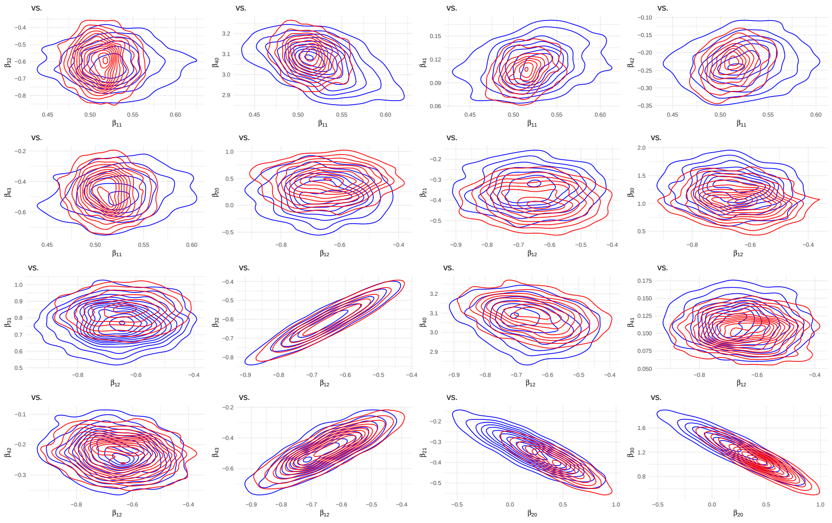

Figure 11. Breast cancer recurrence data: Contour plots of the 2D kernel density estimates for the MCMC samples (blue) and the normal approximation (red).

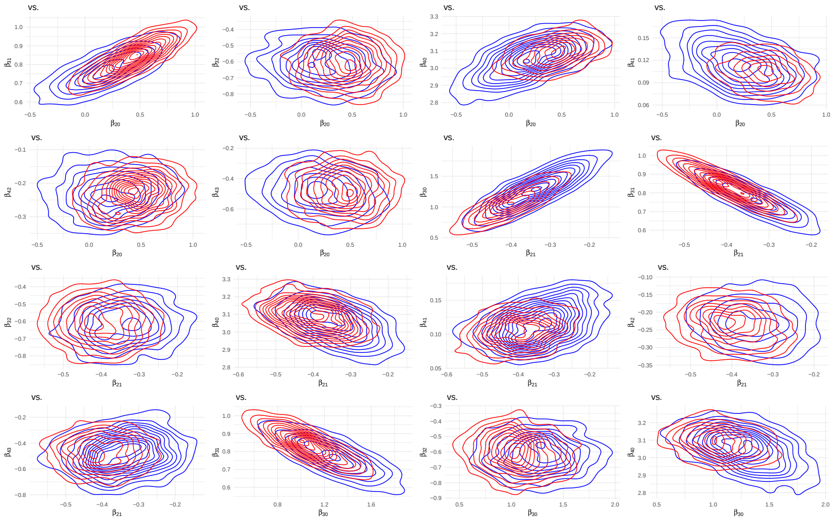

Figure 12. Breast cancer recurrence data: Contour plots of the 2D kernel density estimates for the MCMC samples (blue) and the normal approximation (red).

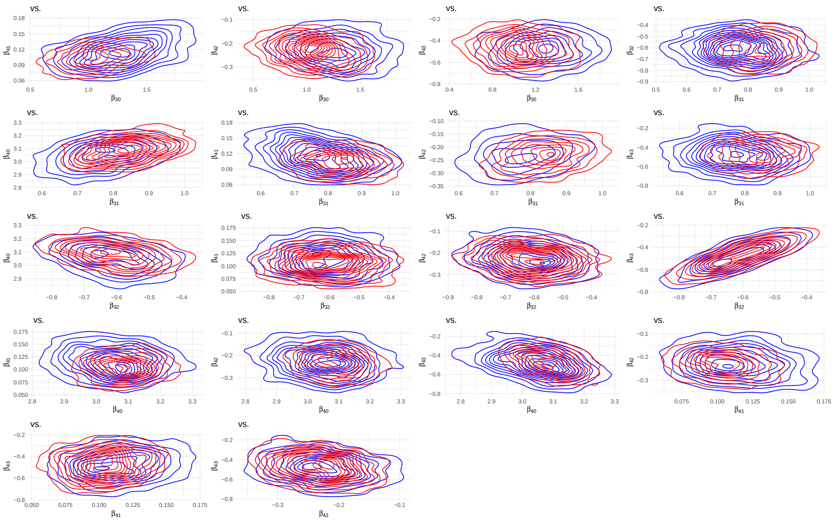

Figure 13. Breast cancer recurrence data: Contour plots of the 2D kernel density estimates for the MCMC samples (blue) and the normal approximation (red).

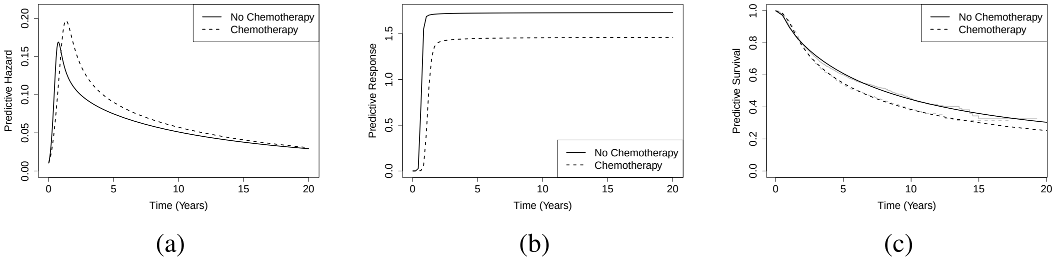

Figure 14. Breast cancer recurrence data (Population): (a) Predictive hazard functions, (b) predictive response functions, and (c) predictive survival functions and Kaplan-Meier estimates.

References

Christen J and Rubio F (2024) Dynamic survival analysis: modelling the hazard function via ordinary differential equations. *Statistical Methods in Medical Research* 33(10): 1768–1782.

Hirsch M, Smale S and Devaney R (2013) *Differential equations, dynamical systems, and an introduction to chaos*. Academic Press.

Hjort N (1992) On inference in parametric survival data models. *International Statistical Review/Revue Internationale de Statistique* : 355–387.

Jackiewicz Z (2009) *General linear methods for ordinary differential equations*. John Wiley & Sons.

Le Cam L and Yang G (1988) On the preservation of local asymptotic normality under information loss. *The Annals of Statistics* : 483–520.

Lehmann E and Casella G (2006) *Theory of point estimation*. Springer Science & Business Media.

Liang X, Livingstone S and Griffin J (2023) Adaptive MCMC for Bayesian variable selection in generalised linear models and survival models. *Entropy* 25(9): 1310.

Nickl R (2013) Statistical theory. *Statistical Laboratory, Department of Pure Mathematics and Mathematical Statistics, University of Cambridge* .

Van der Vaart A (2000) *Asymptotic Statistics*, volume 3. Cambridge University Press.
